# Vision-Related Quality of Life in Primary Angle-Closure Glaucoma Patients with or without Visual Field Dysfunction

**DOI:** 10.1155/2023/9981060

**Published:** 2023-03-20

**Authors:** Pingping Ma, Yingru Liu, Yufang Su, Yajun Yang

**Affiliations:** ^1^Department of Ophthalmology, Dongguan People's Hospital, Dongguan 523000, China; ^2^Department of Glaucoma, Baotou Chaoju Eye Hospital, Baotou 014060, China

## Abstract

**Purpose:**

To evaluate the association between visual-related quality of life (VRQoL) and visual field (VF) loss in patients with primary angle-closure glaucoma (PACG).

**Methods:**

In this case-control study, a total of 79 patients with PACG (with or without VF detects) and 35 healthy controls were included. The patients underwent the 25-item National Eye Institute Visual Functioning Questionnaire (NEI VFQ-25), clinical examination, and VF testing. VF defects were identified by simplified Hodapp's classification. NEI VFQ-25 scores were compared between the three groups.

**Results:**

No significant differences were found in gender, VFQ rating for “composite score” and “color vision” between the three groups. PACG patients with VF loss were most likely to be older and had lower best corrected visual acuity (BCVA), spherical equivalent (SE), mean deviation (MD), and visual field index (VFI), but higher pattern standard deviation (PSD) (all *P* < 0.05). Furthermore, patients with VF loss had significantly lower NVE-VFQ-25 subscale scores for general health, general vision, ocular pain, near activities, distance activities, social functioning, mental health, role difficulties, dependency, driving, and peripheral vision than PACG patients without VF loss and healthy controls (all *P* < 0.05). VFI (*β* = 1.498, *P*=0.003) and MD (*β* = −3.891, *P*=0.016) were significantly correlated with Role Difficulties scores. Additionally, PSD was significantly correlated with Peripheral Vision scores (*β* = −1.346, *P*=0.003).

**Conclusions:**

PACG patients with VF loss reported lower NEI VFQ-25 composite and subscale scores. VF indices including VFI, MD, and PSD were strongly correlated with VRQoL as assessed by NEI VFQ-25; thus, VRQoL may be significantly impacted by glaucomatous VF defects.

## 1. Introduction

Glaucoma is a component of neurodegenerative diseases and is the second leading cause of blindness in the world following cataracts [1, 2]. It was estimated that nearly 80 million people will suffer from glaucoma worldwide by 2020 [3]. Considering its lack of specific symptoms in the early stage, glaucoma brings great psychological stress to patients [4]. Anxiety and depression are the top two common psychological disorders among patients with glaucoma. A previous study indicated that patients with glaucoma were prone to suffer from anxiety and depression [5]. The concept of vision-related quality of life (VRQoL) was introduced in ophthalmology in the 1980s to evaluate the impact of visual impairment on individuals. Previous studies have found that the VRQoL of patients is disrupted by visual field defects [6]. At present, how to maximize the quality of life for glaucoma patients has become a major goal of treatment for the disease. Generally, glaucoma can be divided into two major types: primary angle-closure glaucoma (PACG) and primary open-angle glaucoma (POAG). One of the most common types of disease is PACG, which can cause visual field (VF) damage. The prevalence of POAG is the highest in Africa, while the prevalence of PACG is the highest in Asia [7]. In the early stage of PACG, the VF deficit is absent. Nevertheless, little is known about the association between VF and VRQoL, particularly in PACG patients with relatively normal VF.

Herein, the current study aims to investigate the understanding of VRQoL in PACG patients with or without VF loss and analyze its possible influencing factors on VRQoL.

## 2. Methods

This is a case-control study, which was performed in the Baotou Chaoju Eye Hospital from January to December 2021. This study adhered to the tenets of the Declaration of Helsinki and was approved by the institutional review boards (IRB) of Baotou Chaoju Eye Hospital. When iridotrabecular contact (ITC, ≥2*Q*) resulting in peripheral anterior synechiae (PAS) or raised intraocular pressure (IOP) with no visual field defect or glaucomatous optic nerve damage is found, primary angle closure can be diagnosed. People with glaucoma present glaucomatous optic neuropathy, corresponding retinal nerve fiber layer (RNFL), and VF defects. We recruited adults with newly diagnosed advanced glaucoma, defined according to the extent of visual field loss (Hodapp-Parrish-Anderson classification), in one or both eyes [8]. Informed consent was obtained from all participants at the time of enrollment. Eligibility criteria were as follows: (1) cases were identified as age ≥18 years with a diagnosis of PACG in one or both eyes and (2) healthy controls were identified as without any ocular disorders. Exclusion criteria were as follows: (1) other concomitant eye diseases (such as corneal opacities, severe cataracts, retina, or optic nerve diseases) that can affect visual function and (2) any history of ocular surgery in the past 3 months, the current use of drugs that may cause psychological disorders (such as oral B-receptor blockers), and the cognitive impairment unable to complete the VF examination or questionnaire.

All participants underwent a comprehensive ophthalmological examination, including best corrected visual acuity (BCVA), slit lamp microscopy, indirect ophthalmoscopy (fundoscopy), VF examination, gonioscopy, red-free fundus photography (Canon, Tokyo, Japan) and OCT en face imaging (Cirrus; Carl Zeiss Meditec). BCVA was recorded using the Snellen chart and measured at a distance of 5 m according to standard procedures. VF was examined using a Humphrey visual field analyzer II (Humphrey Instruments, San Leandro, CA) with the standard 24-2 Swedish Interactive Threshold Algorithm strategy (SITA). PACG was defined as eyes with occludable anterior chamber angles and with evidence of glaucomatous optic neuropathy [9]. VF loss was assessed by a mean defect of the visual field (MD) measured in the most recent 3 months prior to candidate selection. The angular width of the RNFL defect was identified by red-free fundus photography (i.e., red-free RNFL defect) and OCT en face imaging (i.e., en face RNFL defect). Only the worse eye was evaluated in each of the patients with PACG, and the right eye of healthy people was included. Demographic characteristics including age, and gender were also collected. Each subject completed the 25-item national eye institute visual function questionnaire (NEI VFQ-25). Subjects were required to complete the questionnaire independently, with the researcher interpreting the questionnaire and providing assistance. For patients with poor visual function, the researchers read the questionnaire to the subjects with a neutral attitude and recorded the choices.

NEI VFQ-25 includes 12 categories, such as general health, general vision, ocular pain, near activities, distance activities, social functioning, mental health, role difficulties, dependency, driving, color vision, peripheral vision, and composite score [10].

### 2.1. Statistical Analysis

SPSS Statistics 20.0 (IBM Corporation, Armonk, NY, USA) was used for data analysis. Continuous variable data were described as mean ± standard deviation (SD). Visual acuity was converted to a logarithm of the minimum angle resolution (logMAR) visual acuity value. One-way analysis of variance (ANOVA) and LSD multiple comparisons were used to analyze the difference in different groups. Multivariate linear regression analysis was used to calculate the relationship between factors and VRQoL. All reported probabilities (*P* values) were two-sided, and *P* value less than 0.05 was considered statistically significant.

## 3. Results

Compared with PACG patients without VF loss as well as healthy control groups, those with VF loss were more likely to be older, having lower BCVA, VFI, and MD values, but having higher PSD values (Table 1).

**Figure 1:**
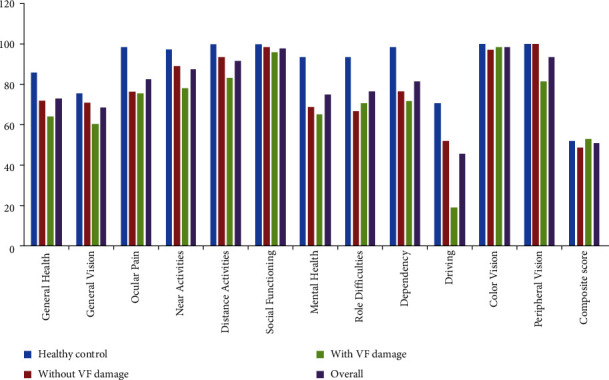
The overall average scores of the NEI VFQ-25.

**Figure 2:**
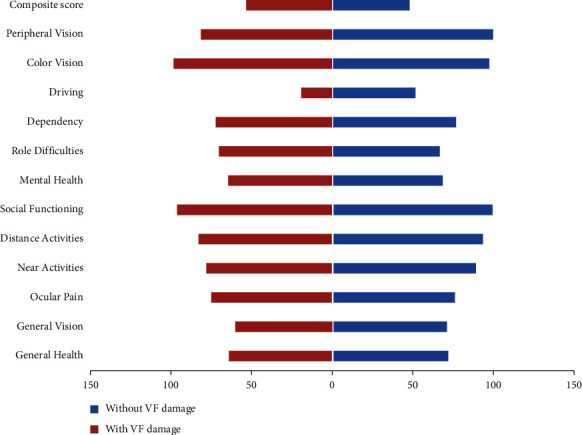
The average scores of the NEI VFQ-25 between PACG patients with and without visual field defects.

**Figure 3:**
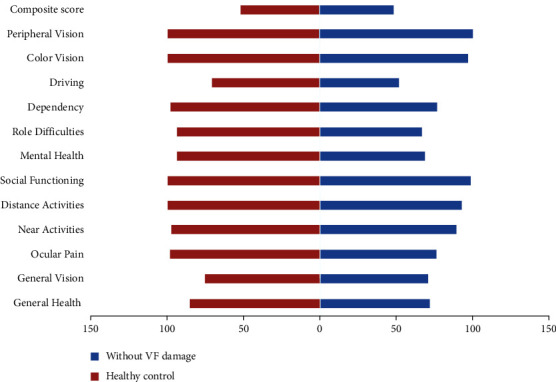
The average scores of the NEI VFQ-25 between PACG patients without visual field defects and healthy controls.

Most categories of VRQoL tended to be better in PACG patients without VF loss and healthy controls than in those with VF loss (all *P* < 0.05); however, no statistical significance of VRQoL scores between group differences were found in “composite score” and “color vision” (all *P* > 0.05, Figure 1). Moreover, ocular pain, mental health, role difficulties, dependency, color vision, and composite score were not significantly poorer in PACG patients with VF loss compared with those PACG patients without VF loss (Figure 2, all *P* > 0.05). Compared with healthy controls, PACG patients without VF loss had lower scores in general health, general vision, ocular pain, near activities, distance activities, social functioning, mental health, role difficulties, and dependency (Figure 3, all *P* < 0.05).

Table 2 shows that age, BCVA, and SE have significant correlations with the VRQoL subscales. Furthermore, VFI (*β* = 1.498, *P*=0.003) and MD (*β* = −3.891, *P*=0.016) were significantly correlated with Role Difficulties scores. Additionally, PSD was significantly correlated with Peripheral Vision scores (*β* = −1.346, *P*=0.003).

## 4. Discussion

Although glaucoma may have an impact on the VRQoL, the changes of VRQoL in patients with or without VF loss are still unclear. The current study indicated that the VRQoL of PACG patients is affected to a certain extent even if their VF tests are normal. Moreover, age, BCVA, SE, and indicators of VF such as VFI, MD, and PSD were correlated with subscales of VRQoL. There is a need to pay more attention to the VRQoL of PACG patients and improving the VF indicators may be a good choice.

Generally, VF tests are an important indicator reflecting the severity of glaucoma [11]. VF is significantly related to VRQoL, that is, the more the glaucoma disease is severe, the worse the VRQoL values [12, 13]. In addition, we also found that even if the PACG patients had no VF defects, their quality of life had some changes, which may be the reason for the decline of BCVA in these PACG patients. To the results of the previous study, patients with vision impairments had worse quality of life than those with normal vision [7]. Moreover, NEI-VFQ-25 scores illustrate different dimensions of the impact on VRQoL, and peripheral vision dysfunction or difficulties in driving were more crucial to glaucoma patients than social factors [14], which were similar to our outcomes. These findings suggest that it is very important to diagnose early glaucoma, intervene early, and protect the visual field and vision of glaucoma patients.

Previously, poor VRQoL was correlated with comorbid eye disease, an anxious personality, poor visual acuity, and MD values. Thus, the evaluation of the impactors on VRQoL in PACG patients required an integrated assessment that included demographic characteristics and ophthalmic biometrics [15]. After adjusting these confounders, age, BCVA, SE, and VF indicators (VFI, MD, and PSD) were independently associated with VRQoL in the current study, which was inconsistent with previous studies [16, 17]. These differences may be because of the study design, ethnicities, social, and cultural disparities between the study populations. A further longitudinal population-based study is still needed to confirm our findings and help improve the VRQoL of patients with PACG.

This study has some limitations. First, the selected influencing factors are limited, and some potential factors such as IOP, and treatment history had not been included. Secondly, all subjects come from a single medical institution, which may cause certain selection biases. Thirdly, although the NEI VFQ-25 has been commonly used in the field of ophthalmic research, it was not specifically designed for PACG patients and VF defects.

## 5. Conclusion

In PACG patients regardless of whether or without VF defects, a decrease in VRQoL has occurred, and the decrease in VRQoL is more pronounced in PACG patients with VF defects compared with PACG patients without VF defects. Overall, the decline in VRQoL in PACG patients was related to age, BCVA decline, SE value, and VF indicators. In conclusion, this study suggests that PACG affects VRQoL scores and is related to the indicators of VF tests. Therefore, we should pay attention to the related indicators of VF, in order to improve the VRQoL of patients with PACG.

## Figures and Tables

**Table 1:** Characteristics of participants with or without visual field damage.

Variables	With VF damage (*n* = 41)	Without VF damage (*n* = 38)	Healthy control (*n* = 35)	*P*
Age (years)	61.79 ± 8.8	56.76 ± 7.24	57.71 ± 7.53	0.013
Gender (female, %)	22 (53.7)	23 (60.5)	18 (51.3)	0.71
BCVA	0.74 ± 0.34	0.96 ± 0.08	0.98 ± 0.06	<0.001
SE	0.58 ± 1.36	0.66 ± 0.85	−0.39 ± 1.51	<0.001
VFI	61.41 ± 32.49	98.13 ± 1.47	97.94 ± 1.91	<0.001
MD	−14.75 ± 10.37	−2.27 ± 1.49	−1.93 ± 1.65	<0.001
PSD	6.58 ± 3.65	2.01 ± 0.81	2.44 ± 1.39	<0.001

BCVA: best corrected visual acuity; SE: spherical equivalent; MD: mean deviation; VFI: visual field index; PSD: pattern standard deviation.

**Table 2:** Correlation coefficients of the association between visual function indexes and NEI-VFQ-25 of total participants.

	Age		BCVA		SE		VFI		MD		PSD	
	*β*	*P*	*β*	*P*	*β*	*P*	*β*	*P*	*β*	*P*	*β*	*P*
General health	−0.37	**0.026**	6.442	0.223	−1.361	0.096	0.181	0.389	−0.083	0.902	−0.157	0.747
General vision	−0.223	0.115	6.976	0.200	0.092	0.912	0.106	0.622	0.364	0.600	−0.024	0.963
Ocular pain	−0.038	0.841	10.902	0.133	−2.439	**0.030**	0.330	0.252	−0.553	0.549	0.027	0.968
Near activities	−0.298	**0.032**	0.695	0.895	−1.092	0.181	0.255	0.227	−0.046	0.946	−0.142	0.771
Distance activities	−0.239	0.068	1.213	0.809	0.302	0.695	0.111	0.577	0.294	0.647	−0.111	0.810
Social functioning	0.101	0.171	10.874	**0.001**	−0.282	0.517	0.041	0.713	−0.130	0.719	−0.272	0.298
Mental health	−0.084	0.722	14.755	0.106	−3.169	**0.025**	0.160	0.658	0.028	0.981	0.020	0.981
Role difficulties	0.003	0.994	−2.189	0.860	−5.215	**0.007**	1.498	**0.003**	−3.891	**0.016**	−0.330	0.774
Dependency	−0.135	0.594	10.411	0.288	−2.927	0.054	0.240	0.538	0.068	0.956	0.502	0.579
Driving	−1.699	**0.004**	2.924	0.895	0.749	0.826	−0.357	0.685	1.647	0.560	−0.876	0.667
Color vision	0.053	0.482	5.060	0.085	−0.531	0.239	0.109	0.349	−0.281	0.452	0.100	0.709
Peripheral vision	−0.367	**0.004**	1.409	0.771	0.800	0.284	0.293	0.131	−0.455	0.462	−1.346	**0.003**
Composite score	−0.276	0.175	22.583	**0.004**	−1.127	0.349	−0.447	0.152	0.896	0.369	0.148	0.837

BCVA: best corrected visual acuity; SE: spherical equivalent; MD: mean deviation; VFI: visual field index; PSD: pattern standard deviation. Bold values indicate statistical significance.

## Data Availability

The data generated or analyzed during this study are included within the article.
